# Effect of Dehydroaltenusin-C12 Derivative, a Selective DNA Polymerase α Inhibitor, on DNA Replication in Cultured Cells

**DOI:** 10.3390/molecules13122948

**Published:** 2008-12-01

**Authors:** Isoko Kuriyama, Takeshi Mizuno, Keishi Fukudome, Kouji Kuramochi, Kazunori Tsubaki, Takeo Usui, Naoko Imamoto, Kengo Sakaguchi, Fumio Sugawara, Hiromi Yoshida, Yoshiyuki Mizushina

**Affiliations:** 1 Laboratory of Food & Nutritional Sciences, Department of Nutritional Science, Kobe-Gakuin University, Nishi-ku, Kobe, Hyogo 651-2180, Japan; E-mails: kuriyama@s.kobegakuin.ac.jp (I. K.); yoshida@nutr.kobegakuin.ac.jp (H. Y.); mizusin@nutr.kobegakuin.ac.jp (Y. M.); 2 Cellular Dynamics Laboratory, Advanced Science Institute, RIKEN, Wako, Saitama, 351-0198, Japan; E-mail: nimamoto@riken.jp (N. I.); 3 Department of Applied Biological Science, Tokyo University of Science, Noda, Chiba 278-8510, Japan; E-mails: j6408638@ed.noda.tus.ac.jp (K. F.); sugawara@rs.noda.tus.ac.jp (F. S.); kengo@rs.noda.tus.ac.jp (K. S.); 4 Graduate School of Life and Environmental Science, Kyoto Prefectural University, Sakyo-ku, Kyoto 606-8522, Japan; E-mails: kuramoch@kpu.ac.jp (K. K.); tsubaki@kpu.ac.jp (K. T.); 5 Graduate School of Life and Environmental Sciences, University of Tsukuba, Tsukuba, Ibaraki 305-8572, Japan; E-mail: usui@sakura.cc.tsukuba.ac.jp (T. U.); 6 Cooperative Research Center of Life Sciences, Kobe-Gakuin University, Nishi-ku, Kobe, Hyogo 651-2180, Japan

**Keywords:** Dehydroaltenusin-C12 (C12), DNA polymerase (DNA-directed DNA polymerase [E.C. 2.7.7.7], pol) α, Enzyme inhibitor, DNA replication, Replication fork uncoupling, Hypotonic shift, Molecule probe, Anticancer drug

## Abstract

Dehydroaltenusin is a selective inhibitor of mammalian DNA polymerase α (pol α) from a fungus (*Alternaria tennuis*). We have designed, synthesized, and characterized a derivative of dehydroaltenusin conjugated with a C12-alkyl side chain (dehydroaltenusin-C12 [C12]). C12 was the strongest pol α inhibitor *in vitro*. We introduced C12 into NIH3T3 cells with the help of a hypotonic shift, that is, a transient exposure of cultured cells in hypotonic buffer with small molecules which can not penetrate cells. The cells that took in C12 by hypotonic shift showed cell growth inhibition. At a low concentration (5 μM), DNA replication was inhibited and several large replication protein A (RPA) foci, which is different from dUTP foci. Furthermore, when C12 was incubated with aphidicolin, RPA foci were not observed in cells. Finally, these findings suggest that C12 inhibited DNA replication through pol α inhibition, and generated single-stranded DNA, resulted in uncoupling of the leading strand and lagging strand synthesis. These findings suggest that C12 could be more interesting as a molecule probe or anticancer agent than aphidicolin. C12 might provide novel markers for the development of antiproliferative drugs.

## Introduction

**Figure 1:**
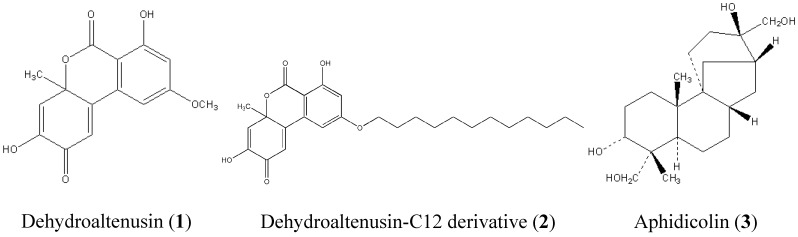
Chemical structures of dehydroaltenusin (**1**), C12 (**2**) and aphidicolin (**3**).

Metazoan organisms are known to contain at least 14 DNA polymerases [1]. Consequently, we have screened for natural compounds that were selective inhibitors of these polymerases [2,3,4]. Selective inhibitors of DNA polymerases must be useful tools in distinguishing DNA polymerases and clarifying their biological and *in vivo* functions [1,5]. Inhibition by the well-known replicative DNA polymerase inhibitor aphidicolin demonstrated that DNA polymerases α, δ and ε (pol α, δ and ε) are essential for replication [6]. Aphidicolin has been very helpful in studying the DNA replication system [7]; however, there have been few previous reports of inhibitors capable of distinguishing among pol α, δ and ε. Among the three DNA polymerases, pol α is not only essential for DNA replication, but is also involved in cell proliferation [1]. Understanding how DNA polymerases are regulated in normal and tumor cells will provide significant novel information on tumor cells and the tumorigenic process and will help to identify novel targets for anticancer therapy. Selective inhibitors of mammalian pol α must be not only molecular tools useful for analyzing polymerases, but should also be considered a group of potentially useful cancer chemotherapy agents. Interestingly, reduced expression of pol α was induced in *S. cerevisiae* genomic instability at fragile sites [8]. Temperature-sensitive tsFT20 has a point mutation in pol α catalytic subunit p180 hypomorphic activity. This cell line leads to chromosomal abnormality [9], because pol α synthesized Okazaki fragment during DNA replication initiation [10,11]. It was surprising that pol α defects increased genomic instability [11]. To understand the cellular physiological function of pol α more in detail, several approaches have been examined so far: 1) microinjection of a specific monoclonal antibody into cells [12], 2) generation of a temperature-sensitive cell line in *S. cerevisiae* [13], and mouse cell lines [14], 3) development of low fidelity pol α enzyme [11,15]. However, low molecular weight chemical inhibitors specific for each DNA replicative polymerase (α, δ, ε) have not identified so far [1]. Dehydroaltenusin (Figure 1(1)) was isolated from a fungus (*Alternaria tennuis*) in the screening for replicative DNA polymerase inhibitors [16]. It was found to be a strong inhibitor of mammalian pol α, but did not influence the activities of mammalian pol δ and ε, or pol α from other vertebrates *in vitro*.

In this report, we chemically synthesized a derivative of dehydroaltenusin, which is stronger pol α inhibitor than dehydroaltenusin, and this compound was conjugated with a C12-saturated alkyl group (i.e., lauric acid) (C12, 2, Figure 1). The inhibitory effects of C12 on pol α activity *in vitro* and DNA replication in cultured cells were investigated, and the role of pol α in DNA replication is discussed.

## Results and Discussion

### Effects of inhibitors on the activities of mammalian pol α

**Figure 2:**
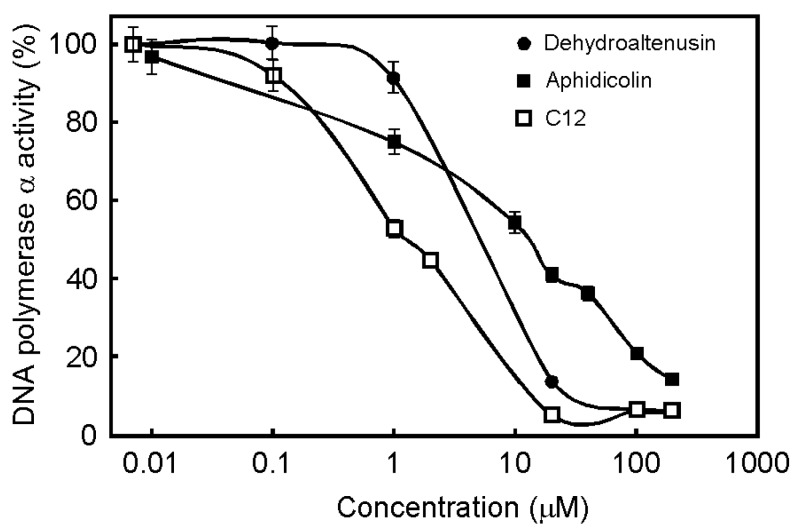
Dose-response curves of dehydroaltenusin, C12 and aphidicolin with DNA polymerase α inhibition. Effects of dehydroaltenusin (closed circle), C12 (open square) or aphidicolin (closed square) on the activities of calf thymus pol α are shown. Amount of enzyme in the assay mixture was 0.05 units. Pol α activity in the absence of the compounds was taken as 100%. Values are shown as the means ± S.E. for three independent experiments.

We reported previously that naturally purified dehydroaltenusin from a fungus (*Alternaria tennuis*) was a specific inhibitor of mammalian pol α. Dehydroaltenusin did not inhibit other mammalian polymerases, such as pol δ, ε, β and γ. We designed novel inhibitors to extend unique properties of dehydroaltenusin, which inhibits only mammalian pol α. We also reported that a longer fatty acid chain is a stronger inhibitor than a short fatty acid chain [2]; however, the solubility of the longer fatty acid chain is poor in aqueous media. Based on these findings, we have designed and chemically synthesized a potent inhibitor, C12, which is a derivative of dehydroaltenusin conjugated with a C12-alkyl group, to analyze the details of DNA replication in mammalian cells. To determine the effect of dehydroaltenusin, C12 and aphidicolin (Figure 1) on pol α *in vitro*, the dose-response curves of these compounds against calf pol α were examined (Figure 2). These inhibitions by dehydroaltenusin, C12 or aphidicolin were dose-dependent. C12 was the strongest pol α inhibitor among these inhibitors, and the IC_50_ value was 1.4 μM. Dehydroaltenusin and aphidicolin also inhibited pol α with IC_50 _ values of 9.9 μM and 14.5 μM, respectively. C12 inhibited pol α stronger than dehydroaltenusin. Aphidicolin inhibited all of the replicative polymerases (pol α, δ and ε), whereas C12 did not inhibit purified human pol δ and human pol ε *in vitro* at all (data not shown). Therefore, we examined their influence on NIH3T3 cell growth.

### Effect of inhibitors on cell growth inhibition

**Figure 3:**
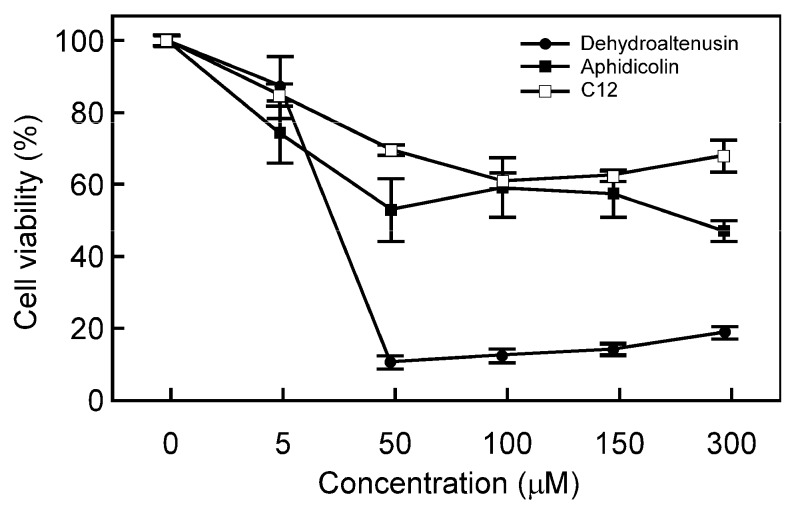
Effect of inhibitors on the cell proliferation of NIH3T3 cells. Dose-dependent inhibition of NIH3T3 cell growth incubated with various concentrations of dehydroaltenusin (closed circle), C12 (open square) or aphidicolin (closed square) for 24 h. Cell proliferation was determined by MTT assay. Cell viability in the absence of the compounds was taken as 100%. Values are shown as the means ± S.E. for five independent experiments.

The inhibitory effect of dehydroaltenusin, C12 and aphidicolin on NIH3T3 cell growth inhibition was examined. Dehydroaltenusin and aphidicolin efficiently inhibited cell growth in a dose-dependent manner (Figure 3). Because C12 hardly inhibited NIH3T3 cell growth, it is suggested that C12 has deficient in incorporation into cells.

### Effect of inhibitors on cell growth by using a hypotonic shift

**Figure 4:**
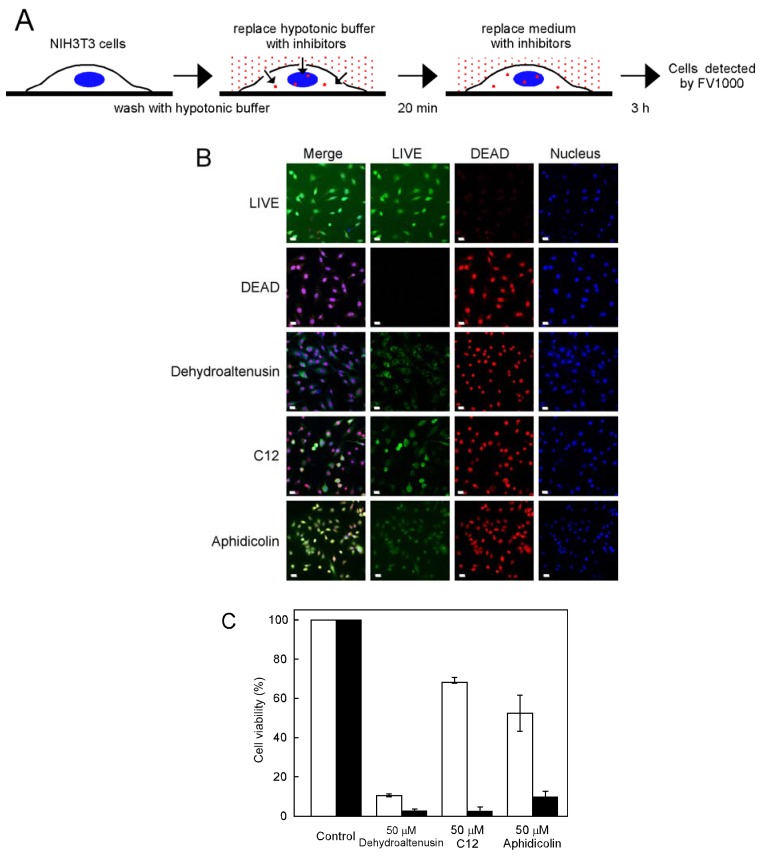
Inhibitors were incorporated into cells using a hypotonic shift. (A) Scheme of the procedure for a hypotonic shift. (B) NIH3T3 cells incubated for 20 min in hypotonic buffer without or with 50 μM dehydroaltenusin, C12 or aphidicolin. The culture medium was then replaced with medium including 50 μM dehydroaltenusin, C12 or aphidicolin for 3 h. Cells were detected using a LIVE/DEAD^®^ Viability/Cytotoxicity Kit. Red and green indicate dead and live cells, respectively. Dead cells were treated with 70% methanol for 30 min. (C) Cell viability using a hypotonic shift (closed bar) or not (open bar) is shown. Live and dead cells were individually counted from at least 200 cells (from each condition). Control was taken as 100%. Values are shown as the means ± S.E. for four independent experiments. All bars indicate 20 μm.

Although C12 was the strongest pol α inhibitor, it did not affect NIH3T3 cell growth, suggesting that C12 is not incorporated into cells; therefore, we investigated whether NIH3T3 cells incorporated C12 with the help of a hypotonic shift, which has been reported as a novel and convenient procedure for introducing hydrophilic molecules into living cells [17] (Figure 4A). Cell viability was determined with the LIVE/DEAD® Viability/Cytotoxicity Kit. This assay kit determines live and dead cells simultaneously, based on the intracellular esterase activity of live cells and plasma membrane integrity of dead cells (Figure 4B). Because this assay kit is very sensitive, rapid and reproducible, it is a suitable assay after hypotonic shift treatment. Cells that incorporated C12 using a hypotonic shift inhibited cell growth with cell viability of 2.2% (Figure 4C). We counted cells stained by ethidium homodimer (Figure 4B) as dead cells. As a result, all inhibitors induced a low survival rate; in particular, C12 markedly inhibited cell growth. There were great differences in cell viability when using a hypotonic shift. This result suggests that a hypotonic shift induces inhibitors into living cells. The nucleus of cells treated with dehydroaltenusin and C12 were stained by Calcein AM. On the other hand, when treated with aphidicolin cells, not only the nucleus was stained but the whole cell by Calcein AM. Aphidicolin was used as a synchronization drug [18]. When aphidicolin was incorporated into cells using a hypotonic shift, it inhibited cell growth. C12 was a stronger cell growth inhibitor than aphidicolin, suggesting that the cause of the cultured cell influence may be the inhibition of pol α activity.

### Effect of C12 on DNA replication

**Figure 5:**
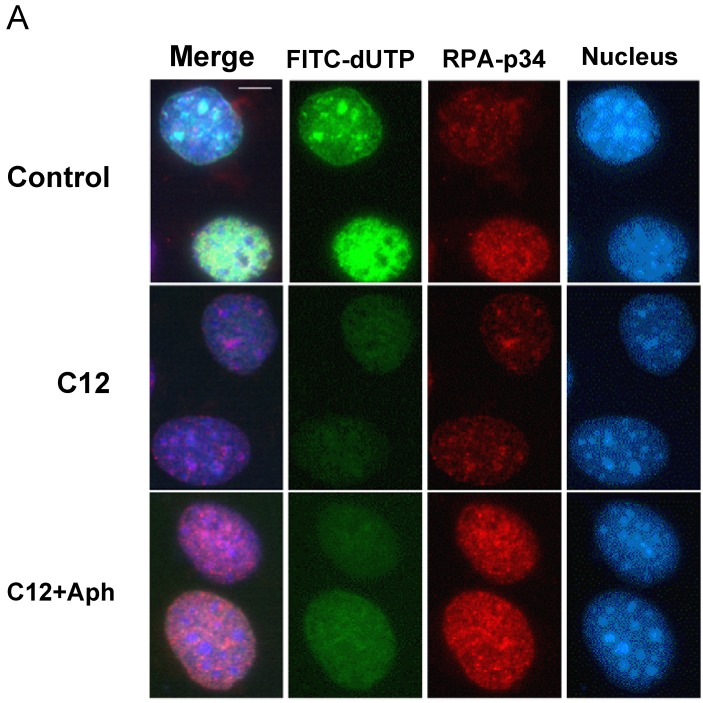

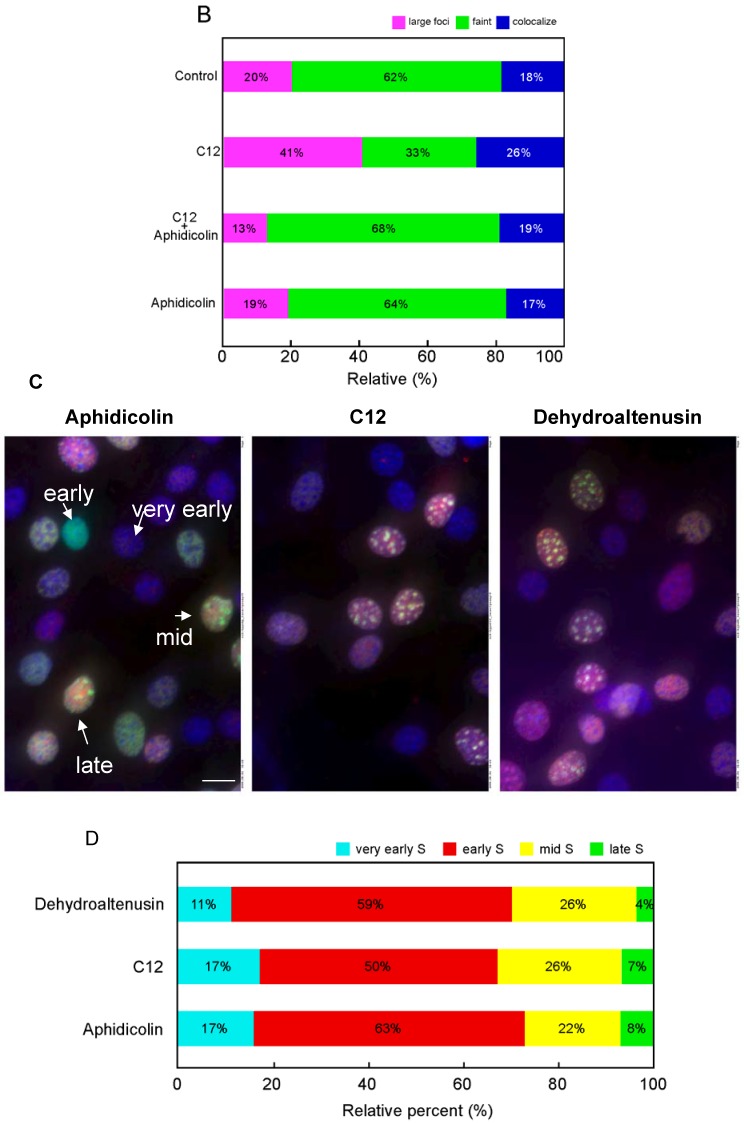
Effect of inhibitors on replication in the nucleus. (A) Immunofluorescent localization of chromatin-bound RPA. NIH3T3 cells was treated by hypotonic shift with FITC-dUTP and C12 or aphidicolin. After 90 min, cells were extracted with cytoskelton buffer containing 0.5% Triton X-100 for 5 min and fixed with 3.7% formaldehyde. RPA-p34 subunit was detected by indirect immunofluorescence with anti-RPA monoclonal antibody and Alexa 594-conjugated anti-mouse antibody. Bar indicates 20 μm. (B) Quantification of the percentage of FITC-dUTP incorporated nuclei showing RPA foci pattern. At least 100 cells were counted. RPA foci were classified into large discrete foci (red), co-localized with FITC (green), and faint (blue). (C) Localization of replication foci. Hypotonic shifttreatedcells were incubated for 2 h, and then chromatin-bound PCNA and pol α p180 were detected. Bar indicates 40 μm. (D) In three independent experiments, 200 cells were counted and the localization of PCNA protein foci in nucleus was classified as very fine granules in the nucleus (very early S), bright foci throughout the nucleus (early S), bright large foci near heterochromatin (mid S) and a few large foci (late S).

To investigate whether inhibition of DNA replication can be observed at lower concentrations using a hypotonic shift, we tested the effect of C12 on DNA replication. Nuclei during the S phase were labeled with FITC-dUTP using a hypotonic shift, and inhibitors were incorporated into cells at the same time. Incorporation of dUTP was significantly reduced with C12, similar to aphidicolin (Figure 5A), suggesting that C12 indeed inhibit DNA replication totally at low concentrations. In addition, treatment of hypotonic shift with C12 resulted in arrested cell cycle progression at the G1/S boundary detected by flow cytometry (data not shown). We then further investigated how C12 inhibited DNA replication and interestingly found that large distinct RPA foci accumulated in C12-treated cell nuclei, implying that single-stranded DNA was generated in the nucleus (Figure 5A, B). Previous studies have shown that RPA, a heterotrimeric single-stranded DNA-binding protein, is involved in the initiation of DNA replication and remains localized to replication forks during elongation in the S-phase [19,20]. Cells treated with C12 showed several RPA foci, different from dUTP foci.

The accumulation of large foci of RPA in the nucleus was canceled in the presence of aphidicolin (Figure 5A, B). We concluded that the effect of C12 on the accumulation of large foci of RPA is derived from pol α inhibition. Manthey *et al*. reported that replication-fork stall and collapse induced RPA foci [21]. Nedelcheva *et al*. reported using a temperature-sensitive mutant of pol α in *S. cerevisiae* when cells were deficient in pol α activity, that mini chromosome maintenance (Mcm) helicase generated single-stranded DNA [22]. Next, we speculated that DNA replication initiation was specifically inhibited by C12 incorporation following pol α inhibition. We examined whether C12 affected the DNA replication of initiation or elongation, observing replication foci by staining proliferation cell nuclear (PCNA) and the catalytic subunit of p180 (Figure 5C, D). Replication foci [23] were classified into typical shapes, and no obvious differences could be detected. To clarify the effect of inhibitors on initiation and elongation more directly, other approaches such as SMARD [24] or DNA fiber analysis [25] are required. In these experiments, we tested the distribution of the localization of chromatin-bound PCNA and the catalytic subunit p180 of pol α after hypotonic shift (Figure 5C). The percentage of cells in very early S and early S, treated with C12, were lower than aphidicolin; C12 and aphidicolin were 67% and 80%, respectively (Figure 5D).

## Conclusions

Here we describe the properties of dehydroaltenusin-C12 derivative (C12, Figure 1(2)), which was designed and synthesized based on dehydroaltenusin and C12-saturated alkyl group, lauric acid. C12 significantly inhibited cell growth by hypotonic treatment due to an indirect effect at high concentrations. These results suggest that C12 can penetrate cells by hypotonic shift and then inhibit DNA replication at low concentrations. Because C12 and dehydroaltenusin inhibited only pol α, not pol δ and pol ε, inhibition of pol α by C12 must lead to fork stalling and uncoupling of leading strand synthesis and lagging strand synthesis. Indeed, we found the accumulation of RPA foci after C12 incorporation into cells, which is a hallmark of single-strand exposure. Finally, we concluded that our unique dehydroaltenusin derivatives introduce replication fork collapse, and uncoupling of leading strand synthesis and lagging strand synthesis, suggesting that C12 is a suitable tool for studying a replication fork progression in mammalian cells. 

Finally, the results in this report suggest that C12 can be a useful tool and a molecular probe for pol α analysis, and the biological and in vivo functions of pol α will be elucidated accurately. Furthermore, C12 has the ability to be effective for use as an anticancer agent based on pol α inhibitory activity.

## Experimental

### General

Nucleotides and chemically synthesized DNA template-primers such as poly(dA) and oligo(dT)_12-18_, and radioisotope reagents such as [^3^H]-dTTP (2'-deoxythymidine 5'-triphosphate) (43 Ci/mmol) were purchased from GE Healthcare Bio-Science Corp. (Piscataway, NJ, USA). All other reagents were of analytical grade and purchased from Nacalai Tesque Inc. (Kyoto, Japan). NIH3T3 cell line (IFO50019), which is a mouse embryotic fibroblast, was supplied by the Health Science Research Resources Bank (Osaka, Japan). 

### Preparation of dehydroaltenusin and dehydroaltenusin-C12 derivative

Dehydroaltenusin (**1**, Figure 1) was prepared from 7-methoxy-2,2-dimethyl-5-[(trifluoromethyl) sulfonyl]-4*H*-1,3-benzodioxin-4-one according to our previously reported method [26,27]. Dehydroaltenusin-C12 derivative (**2**, Figure 1) was synthesized from 7-docyloxy-2,2-dimethyl-5-[(trifluoromethyl)sulfonyl]-4*H*-1,3-benzodioxin-4-one according to the same procedure. The synthetic products were fully characterized by ^1^H- and ^13^C-NMR, infrared spectroscopy (IR), and high resolution mass spectrometry (HRMS), and the data were described previously [28]. 

### Measurement of DNA polymerase activity

Pol α was purified as described previously [29]. For the polymerase activity assay, poly (dA) / oligo (dT)_12-18_ (A/T = 2/1) and [^3^H]-dTTP were used as the DNA template primer and dNTP substrate, respectively. The dehydroaltenusin derivative was dissolved in dimethyl sulfoxide (DMSO) at various concentrations and sonicated for 30 sec. Four microliters of each sonicated sample was mixed with 16 μl of each enzyme (final 0.05 units) in 50 mM Tris-HCl (pH 7.5) containing 1 mM dithiothreitol, 50% glycerol and 0.1 mM EDTA, and kept at 0°C for 10 min. These inhibitor-enzyme mixtures (8 μl) were added to 16 μl of each enzyme standard reaction mixture, and incubated at 37°C for 60 min. Activity without the inhibitor was considered 100%, and the remaining activity at each concentration of the inhibitor was determined relative to this value. One unit of pol activity was defined as the amount of enzyme that catalyzed the incorporation of 1 nmol of dTTP into the synthetic DNA template primer in 60 min at 37°C under the normal reaction conditions for each enzyme [2,30].

### Cell culture and measurement of cell viability

NIH3T3 cells were cultured in Dulbecco's Modified Eagle’s Medium (DMEM) supplemented with 10% calf serum, penicillin (100 units/mL), streptomycin (100 mg/mL) at 37°C in a humid atmosphere of 5% CO_2_/95% air. For the cell viability assay, cells were plated at 1 x 10^3^ into each well of 96-well microplates with various concentrations of dehydroaltenusin, C12 or aphidicolin. Cell viability was determined by MTT assay [31].

### Hypotonic shift treatment

2.5 x 10^4^ cells were plated in each well of an 8-well-chamber slide (Nunc). After incubation for 16 h, the cells were washed with hypotonic buffer (10 mM HEPES. pH 7.4, 30 mM KCl) [17] and then incubated with 50 μM dehydroaltenusin, C12 or aphidicolin in hypotonic buffer for 20 min at 37°C. After 3 h, cell viability was determined by the LIVE/DEAD^®^ Viability/Cytotoxicity Kit (Molecular Probes). In accordance with the manufacturer’s protocol, cells were exposed to Calcein AM (3 μM), which was hydrolyzed by intracellular esterases, and to ethidium homodimer-1 (4 μM), which binds to nucleic acids (The cleavage product of Calcein AM, calcein, produces green fluorescence when exposed to 494 nm light and is used to identify live cells. Bound ethidium homodimer-1 produces red fluorescence when exposed to 528 nm light, allowing the identification of dead cells.) Dead cells were treated with 70% methanol for 30 min. Culture dishes were stained and cell viability was examined under a confocal laser-scanning microscope (Olympus FV1000; Olympus).

### Immunofluorescence microscopy

Eight-well chamber slides (Nunc.) were pre-treated with acid-collagen (Koken) (1 μg/mL) for 2 h at 37˚C and 3 x 10^4^ cells were seeded per well and incubated with 3 mM thymidine for 16 h at 37°C. The cells were washed two times with medium without thymidine and hypotonic buffer, respectively, and incubated with hypotonic buffer with 10 µM FITC-dUTP (Roche), or 5 µM aphidicolin, 5 µM dehydroaltenusin, and 5 µM C12, respectively, for 10 min at 37°C, and incubated for 90 min with medium at 37°C. The cells were pre-extracted with 0.5% Triton X-100 in cytoskeleton buffer (10 mM PIPES-KCl, pH 6.8, 100 mM KCl, 300 mM sucrose, 2 mM MgCl_2_, 1 mM EGTA, 1 x complete protease inhibitors (Roche), 1 mM DTT and 0.25 mM PMSF 5 min on ice, and then fixed with 3.7% formaldehyde in PBS on ice 10 min, incubated with 50, 75 and 95% ethanol for 5 min each and image IT (Invitrogen) at RT 15 min, and 5% normal goat serum in PBS 15 min at RT. Cells were incubated with anti-p180 [32] and anti-PCNA (Sigma) or anti-RPA p34 (Upstate) antibodies and Alexa488- conjugated anti-mouse IgG secondary antibody and Alexa594-conjugated anti-rabbit IgG secondary antibody (Invitrogen). DNA was stained with Hoechst33258. In three independent experiments, 200 cells were counted and the localization of PCNA protein foci in nucleus was classified as very fine granules in the nucleus (very early S), bright foci throughout the nucleus (early S), bright large foci near heterochromatin (mid S) and a few large foci (late S). For RPA foci observation, 100 FITC-dUTP incorporated cells were analyzed and classified into three patterns as 1) very faint, 2) colocalized with FITC-dUTP, and 3) large discrete foci [19].
